# Shedding Light on the Role of the Skin in Vaccine-Induced Protection against the Malaria Sporozoite

**DOI:** 10.1128/mBio.02555-18

**Published:** 2018-12-11

**Authors:** Johanna Patricia Daily

**Affiliations:** a Albert Einstein College of Medicine, Department of Medicine (Infectious Diseases), Bronx, New York, USA

**Keywords:** antimalarial vaccine, malaria immunity, malaria pathogenesis

## Abstract

The most advanced vaccine against Plasmodium falciparum malaria, RTS,S/AS01, provides partial protection in infants and children living in areas of malaria endemicity. Further understanding its mechanisms of protection may allow the development of improved second-generation vaccines. The RTS,S/AS01 vaccine targets the sporozoites injected by mosquito vectors into the dermis which then travel into the blood stream to establish infection in the liver. Flores-Garcia et al. (Y. Flores-Garcia, G. Nasir, C. S. Hopp, C. Munoz, et al., mBio 9:e02194-18, 2018, https://doi.org/10.1128/mBio.02194-18) shed light on early protective responses occurring in the dermis in immunized animals. They demonstrated that immunization impairs sporozoite motility and entry into blood vessels. Furthermore, they established that challenge experiments performed using a dermal route conferred greater protection than intravenous challenge in immunized mice. Thus, the dermal challenge approach captures the additional protective mechanisms occurring in the dermis that reflect the natural physiology of infection. Those studies highlighted the fascinating biology of skin-stage sporozoites and provided additional insights into vaccine-induced protection.

## Abstract (precis)

The most advanced vaccine against Plasmodium falciparum malaria, RTS,S/AS01, provides partial protection in infants and children living in areas of malaria endemicity. Further understanding its mechanisms of protection may allow the development of improved second-generation vaccines.

## COMMENTARY

Plasmodium falciparum infects millions of people each year and is associated with high mortality rates (1). An effective malaria vaccine would provide a breakthrough intervention to reduce disease worldwide and provide an additional weapon toward eradication. The most advanced vaccine, RTS,S/AS01, includes a recombinant malarial protein (circumsporozoite protein [CSP]) and provides partial protection against clinical malaria in children (vaccine efficacy [VE], 36.3%; 95% confidence interval [CI], 31.8% to 40.5%) and in young infants (VE, 25.9%, 95% CI, 19.9% to 31.5%) (2). The RTS,S/AS01 vaccine is now being tested in a multicountry vaccine trial under real-world conditions. Improving the efficacy of RTS,S/AS01 and informing the development of more-robust vaccines would allow a greater impact of these interventions on global health.

RTS,S/AS01, along with other malaria vaccines under development, targets the sporozoite stage, which is the stage of initial human infection where the number of organisms is low, thus representing an ideal target. Sporozoites are inoculated into the dermis by a female *Anopheles* mosquito during a blood meal and then enter the circulation and invade the liver to establish an infection. This initial stage of infection involving the dermis was recognized in early reports; however, few of the details regarding the journey of the sporozoites through the dermis and how they invade blood vessels to reach the liver are known (3, 4). Similarly, the effect of antisporozoite antibodies induced by RTS,S/AS01 or sporozoite immunization on the dermal stage of infection is poorly understood. The study by Flores-Garcia et al. examined the effect of antisporozoite antibodies and sporozoite immunization on sporozoite challenge during this black box stage of dermal infection.

## DERMAL ANTISPOROZOITE IMMUNE RESPONSES ARE IMPORTANT FOR PROTECTION

Surrogate markers of antisporozoite vaccine efficacy typically include measurements of blood antibodies and cell-mediated immunity to CSP (5). Flores-Garcia et al. examined the effect of immunization on parasite movement during the earliest stage of infection by measuring sporozoite motility in the skin and infection of dermal blood vessels (6). They demonstrated that both immunization with irradiated sporozoites and the activity of anti-CSP antibodies impaired sporozoite motility upon sporozoite challenge compared to the results seen with naive mice. They then showed that the impairment of motility is specific to immune responses to CSP. Using intravital imaging of fluorescently labeled P. berghei sporozoites, they measured the complex movements of sporozoites and quantified the reduction in sporozoite motility, displacement, and speed and changes in trajectories in vaccinated mice. These effects on sporozoite movement are associated with reduced blood vessel invasion and liver-stage infection. Thus, they demonstrated that antibodies against CSP are acting in the dermis.

Building on this observation, they tested whether sporozoite challenge in the dermis compared to the standard intravenous challenge model results in different levels of protection in the animal model. This is an important experiment, as the standard method to test antisporozoite vaccine efficacy is typically through intravenous challenge. They demonstrated that protection against liver-stage infection is more pronounced in challenges with sporozoites through the skin than in intravenous challenge. Thus, that paper highlights the importance of studying immune effects on sporozoite biology during the dermal stage and that vaccine efficacy studies performed via dermal challenge may provide more physiologic data on vaccine performance.

## BUT HOW DO DERMAL IMMUNE RESPONSES TO THE SPOROZOITE PROVIDE PROTECTION?

Attenuated sporozoites inoculated into the dermis provide protection upon sporozoite challenge, but the contribution of skin-stage immune responses to this protection is unclear. The dermis acts as an immune organ, rich with blood vessels and lymphatics able to serve as portals for immune cells (7). The skin hosts tissue-resident phagocytes, antigen presenting cells, mast cells, T lymphocytes, innate lymphoid cells, and antibodies. What role do these play in vaccine-mediated protection? Previous research has produced mixed results. Some studies showed that the intravenous route of administration of attenuated sporozoites provides higher protective efficacy than intradermal inoculation due to induction of immune suppressive responses during intradermal infection (7). Other studies found no difference in protective efficacy levels between intravenous and intradermal injection (8). One comprehensive analysis of host responses, carried out in an animal model after the dermal inoculation of sporozoites (9), found that neutrophils and inflammatory monocytes were recruited to the skin and the draining lymph node and that a Th1 immune response was generated rapidly after infection. In addition, live parasites were found inside CD11b^+^ cells, suggesting a potential reservoir of infection. Furthermore, parasite-specific CD8^+^ T cells were found in the draining lymph node, consistent with prior studies highlighting a role of draining lymph nodes in the generation of protective CD8^+^ T cell responses (10). Further studies of dermal immune responses to the sporozoite stage will provide new knowledge on the basic immunology of malaria infection, determine the ideal route for vaccination, and provide additional readouts of efficacy for testing sporozoite-stage vaccines.

Flores-Garcia et al. have contributed to our understanding of the dermal immune response by showing that the neutralizing capacity of anti-CSP antibodies is greater after challenge in the skin site than in the blood circulation. Although the basis for this difference was not established, perhaps the sporozoite is more vulnerable to antibody-mediated immunity in the dermis than in the circulatory system. It will be interesting to compare the various antisporozoite vaccines presently in development with respect to their ability to reduce sporozoite motility in the dermis. How do immune responses in the dermis alter parasite mobility to reduce the establishment of infection? Studies have shown that the presence of immune sera results in a precipitate that forms around the sporozoite *in vitro* and can result in sporozoite lysis (11). Whether this occurs *in vivo* is unknown. Other intriguing questions include the following. (i) What dermal cellular host responses are associated with protection? Are there specific functional properties of anti-CSP antibodies in the tissue that contribute to protection in the skin and are other dermal cofactors involved? (ii) How do sporozoites detect and invade blood vessels? Answering this latter question could provide an additional target of intervention. Sporozoites are able to traverse liver sinusoids by multiple mechanisms, targeting Kupffer cells or endothelial cells, and perhaps similar mechanisms allow traversal of dermal blood vessels to enter the circulation (12). (iii) When sporozoites enter the bloodstream, do dermal-stage responses impact establishment of liver-stage infection and protection against asexual-stage infection? Shedding light on the black box of the dermal-stage infection may provide new information on skin-stage biology and the role of dermal-stage host responses in infection outcomes.

## CONCLUDING REMARKS

The development of the RTS,S/AS01 vaccine has been a major breakthrough for the control of P. falciparum infections and has saved countless lives. Improving its efficacy and informing novel vaccines that target the sporozoite phase requires a deeper understanding of the protective immune processes occurring at the site of infection, where Flores-Garcia et al. demonstrated that potent protective mechanisms occurs after immunization. Their studies shed further light on the mysterious movements of the sporozoite during its journey through the skin and into the circulation and open the door for additional basic discoveries revealing the biology of the skin stage. Finally, their studies highlighted the need to assess dermal-stage protection during challenge experiments, in addition to the standard surrogate markers of protection, for the evaluation of antisporozoite vaccine effectiveness.
